# Mild Acid-Assisted Separation and Closed-Loop Monomer-Level Regeneration of Polyamide 6 from Waste Wool/PA6 Carpet Blends

**DOI:** 10.3390/polym18151800

**Published:** 2026-07-23

**Authors:** Guohu Wang, Shimu Yu, Fengzhuang Liu, Guodong Liu, Chengsheng Zhang, Hongxin Zhang

**Affiliations:** 1 School of Chemical Engineering, Qinghai University, Xining 810016, China; 2 Shengyuan Carpet Group Co., Ltd., Xining 810016, China

**Keywords:** wool/PA6 carpet blends, mild acid-assisted separation, closed-loop recycling, microwave-assisted depolymerization, caprolactam regeneration

## Abstract

Efficient component separation is a prerequisite for high-value recycling of blended textile waste. Herein, a two-stage metal-free recycling process for wool/polyamide 6 (PA6) carpet waste is developed, combining mild acid-assisted selective separation with monomer-level PA6 regeneration. Acetic acid pretreatment at 80 °C removes surface dyes, followed by formic acid treatment for selective PA6 dissolution. Both acids are efficiently recyclable via low-temperature rotary evaporation, with an average PA6 recovery yield over 75% across five consecutive cycles. Recovered wool preserves its original scale structure and tensile strength comparable to virgin fibers, and reclaimed PA6 maintains its macromolecular and crystalline structure, verifying the mildness of the separation protocol. The recovered PA6 is further depolymerized via a microwave-assisted acetic anhydride/organobase system, where the phosphazene base *^t^*BuP_4_ achieves a maximum *N*-acetyl-*ε*-caprolactam yield of 74.6%. Depolymerization efficiency is governed by catalyst basicity, nucleophilicity, and acetic anhydride-mediated polyamide backbone activation. After deacetylation, the resulting *ε*-caprolactam is repolymerized with the same *^t^*BuP_4_ catalyst, producing regenerated PA6 with chemical structure and thermal properties nearly identical to virgin PA6.

## 1. Introduction

Blending natural and synthetic fibers is a well-established approach to producing high-performance textiles by combining complementary material properties [1]. As a classic natural fiber, wool is prized for its exceptional elasticity and soft tactile qualities, making it indispensable in the manufacturing of high-quality carpets. The incorporation of polyamide (PA) further endows wool fabrics with outstanding abrasion resistance and mechanical robustness [2], establishing wool/PA blends as the predominant material for commercial indoor and public facility carpets worldwide. Despite their favorable performance, such carpets generally have a service lifespan of less than 20 years [3]. As of 2022, this rapid product turnover contributes to an estimated 5 million tons of post-consumer carpet waste generated each year globally, with less than 5% recycled for high-value reuse and over 90% disposed of in landfills or incinerated [3,4]. While wool is biodegradable with relatively mild long-term ecological impacts [5,6], the non-biodegradability of PA makes conventional disposal practices not only a substantial waste of finite petrochemical resources but also a persistent threat to soil and groundwater ecosystems via microplastic leaching and chemical additive release [7,8].

Efficient fractionation of individual components is the fundamental prerequisite for high-value recycling of such multi-component-blended waste [9]. Existing recycling approaches for wool/PA-blended waste remain dominated by mechanical routes: waste fabrics are shredded, carded, and reprocessed into nonwoven mats for thermal insulation, sound absorption, or furniture filling [10]. Although this process is technically mature, severe mechanical shearing causes irreversible fiber breakage and polymer chain degradation, and the resulting products are suitable only for low-end applications with very limited added value [11,12]. Improved physical separation technologies, including electrostatic separation and air classification, avoid thermal damage to fibers during processing but generally experience low separation accuracy for finely blended fibers and high energy consumption, making them difficult to scale up for carpet waste treatment [13]. Chemical or biochemical routes provide targeted separation pathways, but traditional strong acid/alkali treatments inevitably cause irreversible structural damage to the retained component [14,15,16]. Keratinase-based methods exhibit high specificity for wool degradation [17], yet their large-scale industrial application is constrained by long reaction times and prohibitively high enzyme costs.

In recent years, selective dissolution–precipitation (SDP) has emerged as a promising pretreatment technology for separating blended textiles. For PA-containing waste streams, various solvent systems have been explored, including glycols, calcium chloride–alcohol solutions, ionic liquids, and carboxylic acids [18]. Among these, diol-based solvents exhibit excellent PA selectivity but require high dissolution temperatures, leading to high energy consumption and difficulty in solvent recovery [19]. Ionic liquids dissolve PA through hydrogen-bonding and Lewis acid–base interactions with amide groups [20], yet their high cost and complex regeneration processes hinder industrial scalability [21,22]. The calcium chloride–alcohol system, as a milder alternative, is the only solvent platform currently applied to wool/PA-blended fiber separation. However, residual calcium ions are extremely difficult to remove from regenerated PA, disrupting reformation of intermolecular hydrogen bonds and reducing crystallinity and mechanical properties [23,24]. By comparison, carboxylic acids such as formic acid (HCOOH) represent another well-documented class of PA solvents, offering moderate cost, easy recovery via distillation, and good solubility at low-to-moderate temperatures [25,26]. Nevertheless, their feasibility and separation performance in wool/PA-blended systems remain largely underexplored. More critically, although mature depolymerization technologies for converting polyamide 6 (PA6) into *ε*-caprolactam (CPL) have been developed [7], no continuous, integrated process combining fiber separation, PA depolymerization, and monomer purification has been reported for wool/PA6-blended carpet waste to date, leaving a critical gap in closed-loop recycling of blended carpet waste.

Against this background, this work develops a two-stage closed-loop recycling strategy for wool/PA6-blended fibers derived from post-production carpet waste. In the first stage, a mild sequential acid treatment protocol is designed for component separation: acetic acid (AcOH) is used as a pretreatment to remove physically adsorbed dyes and surface impurities, followed by selective dissolution of PA6 using HCOOH while preserving the structural integrity of wool fibers. This metal-free dissolution system eliminates the metal-ion residue problem inherent in conventional calcium salt-based methods. In the second stage, a microwave-assisted acetic anhydride/organobase catalytic system is employed to efficiently depolymerize the recovered PA6, yielding high-purity CPL monomers via deacetylation. The obtained CPL can be directly repolymerized into PA6 with chemical structure and thermal properties comparable to those of virgin materials. This work presents a new technical route for the closed-loop recycling of wool/PA-blended textile waste under mild, metal-free conditions, offering a methodological reference for treating other PA-containing blended waste streams.

## 2. Materials and Methods

### 2.1. Materials and Reagents

Waste wool/PA6-blended carpets, monochromatic wool/PA6-blended fibers dyed with C.I. Acid Blue 317 and C.I. Acid Black 207, as well as undyed pure PA6 fibers and pure wool fibers, were provided by Shengyuan Carpet Group Co., Ltd., Xining, China. The waste carpets are post-production offcuts of commercial Axminster woven carpets. The pile layer (7 × 12 stitching density, 980 g m^−2^ pile weight) is composed of wool/PA6-blended yarns at a wool/PA6 mass ratio of 80:20. The blended yarns have a linear density of 660 tex/2. Specifications of raw fibers are as follows: wool fibers have a dominant staple length of 3–5 inches, an average diameter of 35 μm, and a moisture regain of 13%. PA6 fibers have a cut length of 10–11 cm, a diameter of 40 μm, and a moisture regain of 4%. Prior to use, all fiber samples were washed three times with deionized water, then dried in a vacuum oven at 60 °C for 12 h, and finally cut into 5–10 mm segments. This chopping step ensures sufficient acid penetration into tightly twisted carpet yarns and improves mass transfer efficiency during the dissolution process, which is a standard pretreatment widely adopted in textile waste recycling [10,18].

Acetic acid (AcOH; Energy Chemical, 99%, Shanghai, China), formic acid (HCOOH; Energy Chemical, 99%, Shanghai, China), chlorobenzene (Energy Chemical, 99%, Shanghai, China), thiourea dioxide (Energy Chemical, 99%, Shanghai, China), sodium dodecyl sulfate (SDS; Energy Chemical, 99%, Shanghai, China), acetic anhydride (Ac_2_O; Energy Chemical, 98.5%, Shanghai, China), monoethanolamine (MEA; Energy Chemical, 99.5%, Shanghai, China), 1,5,7-triazabicyclo[4.4.0]dec-5-ene (TBD; TCI, 98%, Shanghai, China), 1,8-diazabicyclo[5.4.0]-7-undecene (DBU; TCI, 98%, Shanghai, China), 4-dimethylaminopyridine (DMAP; TCI, 98%, Shanghai, China), triethylamine (TEA; Energy chemical, 99%, Shanghai, China), pyridine (Py; TCI, 99.9%, Shanghai, China) and 1-*tert*-Butyl-4,4,4-tris(dimethylamino)-2,2-bis[tris-(dimethylamino)-phosphoranylidenamino]-2*λ*^5^,4*λ*^5^-catenadi(phosphazene) (*^t^*BuP_4_; Aldrich, 0.8 M solution in *n*-hexane, Shanghai, China) were used as received without further purification.

### 2.2. Fiber Pretreatment and Separation of Wool and PA6

Shredded carpet fibers (10.0 g) were heated in 300 mL AcOH at 80 °C for 5 h with constant stirring (fiber to acid ratio = 1:30, *w*/*v*). After pretreatment, the mixture was hot-filtered to collect the purified fibers, which were then washed twice with 50 mL of AcOH. The filtrate was collected for AcOH recovery via rotary evaporation. The pretreated fibers were subsequently immersed in 300 mL of HCOOH at 60 °C for 5 h to selectively dissolve PA6. Insoluble wool residues were removed by filtration and washed twice with 50 mL of HCOOH. The HCOOH solution was concentrated to approximately 10 mL under reduced pressure, and then slowly added to excess deionized water to precipitate PA6 as a nearly white powder. The precipitated PA6 was collected by filtration, washed three times with deionized water, and dried in a vacuum oven at 80 °C for 12 h.

The PA6 recovery yield was calculated using the following Equation (1):

(1) $$Recovery\;yield\left(\%\right)=\frac{m_{recovered\;PA6}}{m_{theoretical\;PA6}}\;\times 100\%$$

where *m*_theoretical PA6_ is the theoretical mass of PA6 in the feedstock, calculated from the wool/PA6 mass ratio (80:20) of the blended fibers.

Acid recycling tests: Five consecutive separation and recycling cycles were conducted for both AcOH and HCOOH solvents to evaluate the economic feasibility and separation stability of the approach. At the end of each cycle, the acid solutions were recovered by rotary evaporation and reused in the subsequent separation experiment, with the bath ratio kept constant at 30:1 throughout all cycles, based on the volume of recovered AcOH. For each cycle, the actual volume of the recovered acids was recorded to calculate acid recovery efficiency, and the mass of the separated PA6 and wool fibers was noted to calculate their respective recovery rates.

### 2.3. Depolymerization of Recovered PA6

The depolymerization of recovered PA6 was performed according to the literature with minor modifications [27]. A typical procedure for depolymerization is as follows: Recovered PA6 powder (0.10 g, 8.85 mmol based on repeating unit, 1.0 equiv.), Ac_2_O (12.5 mL, 15.0 equiv.) and *^t^*BuP_4_ (562.0 μL, 0.45 mmol) were charged into a 50 mL microwave vial containing a magnetic stir bar in a glovebox. Microwave reactions were performed in a microwave synthesizer (XH-800SP, Beijing Xianghu Technology Development Co., Ltd., Beijing, China). The mixture was heated up to 230 °C at a heating rate of 10 °C min^−1^ and maintained at this temperature for 30 min, with the pressure monitored to remain below 4 MPa. After cooling to room temperature, the dark crude solution was filtered through a short pad of Celite to remove any insoluble residues that may be present, and most of the excess Ac_2_O was subsequently removed by rotary evaporation at 60 °C. The remaining dark viscous residue was rinsed into a distillation apparatus with small portions of AcOH, and pure *N*-acetyl-ε-caprolactam (AcCL) was collected via distillation under reduced pressure as a yellow viscous liquid.

### 2.4. Deacetylation of N-Acetyl-ε-Caprolactam

High-purity CPL was synthesized through transamidation between AcCL and MEA following a modified literature procedure [22]. Briefly, AcCL (1.0 g, 6.4 mmol, 1.0 equiv.), MEA (0.43 g, 6.8 mmol, 1.05 equiv.) and toluene (2.0 mL, [AcCL]_0_ ≈ 3.0 M) were charged into a 50 mL three-necked flask equipped with a reflux condenser and a nitrogen inlet. The mixture was heated to 80 °C and stirred under a nitrogen atmosphere for 2.5 h. Upon cooling to room temperature, the solvent was removed under reduced pressure, and the crude product was purified using silica gel column chromatography (CH_2_Cl_2_:methanol = 9:1). The obtained CPL was further recrystallized from petroleum ether/ethyl acetate (4:1) to afford colorless crystals.

### 2.5. Repolymerization of Regenerated ε-Caprolactam

Following a modified literature procedure [28,29], purified regenerated CPL (5.0 g, 44.0 mmol) was transferred into a 100 mL flask equipped with a magnetic stir bar and an argon inlet. After thoroughly purging the atmosphere, the flask was heated to 100 °C in an oil bath to melt the CPL monomer, and *^t^*BuP_4_ (0.25 mol% relative to CPL) was added as the catalyst. The mixture was stirred at 120 °C for 30 min to activate the monomer, after which AcCL (68 mg, 0.44 mmol) was introduced as the initiator. The reaction proceeded at 180 °C until a significant increase in viscosity was observed (approximately 3 h), and then quenched by cooling to room temperature. The crude polymer was dissolved in HCOOH, and the solution was added to excess deionized water to precipitate a white solid.

### 2.6. Characterization Methods

Size exclusion chromatography (SEC) was conducted in HFIP at 40 °C using two identical PL HFIgel columns (9 μm) at a flow rate of 1.0 mL min^−1^. Calibration was done with a series of narrowly dispersed poly(methyl methacrylate) standards to obtain apparent number-average molar mass (*M*_n,SEC_) and molar mass distribution (*Ð*) of the polymers. ^1^H NMR was recorded at room temperature (RT) on a Bruker AV600 NMR spectrometer (Bruker BioSpin GmbH, Rheinstetten, Germany) using DMSO-*d*_6_ as the solvent and tetramethylsilane as an internal standard. X-ray Diffraction (XRD) was measured on a Rigaku D/Max 2500 instrument (Rigaku Corporation, Tokyo, Japan) using a Cu K*α* radiation source (*λ* = 1.5406 Å) in the scanning range from 10° to 80° with a step size of 5° min^−1^. Fourier transform infrared spectroscopy (FTIR) was recorded on a Nicolet iN10 FT-IR spectrometer (Thermo Fisher Scientific, Waltham, MA, USA) in the range of 4000–400 cm^−1^ at 25 °C. The surface morphology of isolated wool fibers was studied using a JSM-6610LV SEM (JEOL Ltd., Tokyo, Japan). Samples were gold-sputtered for 30 s to enhance conductivity and then imaged at 1.00 kV accelerating voltage using a secondary electron detector. Tensile tests of wool yarns were conducted on a Zwick/Roell Z1.0TN universal testing machine (ZwickRoell GmbH & Co. KG, Ulm, Germany) in accordance with ASTM D882 at a gauge length of 10 cm and a stretching rate of 25 mm·min^−1^. At least 10 individual wool yarns were tested per sample to ensure statistically reliable results. Differential scanning calorimetry (DSC) measurements were performed on a METTLER TOLEDO DSC3 system (Mettler-Toledo International Inc., Greifensee, Switzerland) in a nitrogen flow. The sample was quickly heated to 300 °C, kept at this temperature for 10 min to remove thermal history, cooled to −80 °C at a cooling rate of 10 °C min^−1^, and then heated again to 300 °C at a heating rate of 10 °C min^−1^. Thermogravimetric analysis (TGA) was conducted on a NETZSCH TG209F3 thermal analyzer (NETZSCH-Gerätebau GmbH, Selb, Germany) under a nitrogen atmosphere at a heating rate of 10 °C min^−1^ from 25 to 800 °C.

## 3. Results and Discussion

### 3.1. Mild Acid-Assisted Separation of Wool and PA6

**Scheme 1:**
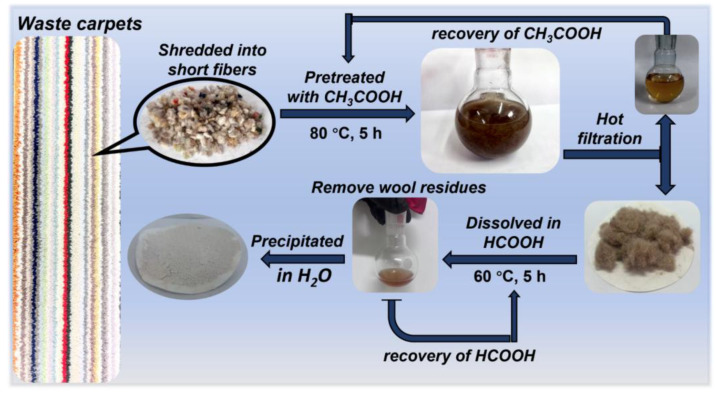
Schematic illustration of the acid-assisted sequential separation process for waste wool/PA6 carpet fibers, including AcOH decontamination, HCOOH selective dissolution of PA6, and water-induced reprecipitation for PA6 recovery.

The acid-assisted sequential separation process is depicted in Scheme 1. This strategy capitalizes on the differential chemical stability of wool and PA6 in acidic environments. Specifically, wool fibers maintain chemical stability under mildly acidic conditions but are susceptible to irreversible structural degradation in alkaline environments. As a result, industrial wool processing, particularly dyeing, is commonly performed in acidic dye baths where AcOH is used to modulate pH [30]. Leveraging this unique chemical susceptibility, AcOH serves as a gentle pretreatment to remove physically adsorbed dyes without damaging the wool structure. In contrast, HCOOH is specifically chosen for PA6 dissolution, given that PA6 exhibits high solubility in HCOOH [31], allowing for the efficient and selective solvation of PA6 components. Following the two-step acid treatment, wool fibers are filtered out, and the isolated solid PA6 powder is collected via water-induced reprecipitation. Both acids can be largely recovered through rotary evaporation and reused, thereby minimizing the environmental impact of the process.

To confirm the selectivity of this separation protocol, a control experiment was conducted using pure wool and pure PA6 fibers pretreated with AcOH. Post-AcOH treatment, the mass loss of both fibers was minimal (both less than 1%), indicating that the pretreatment did not cause significant dissolution of the wool or PA6 fibers (Figure S1a). Conversely, in the blend, the PA6 component dissolved quickly in HCOOH, and most of the dissolved PA6 was recovered as a solid through reprecipitation. The average PA6 recovery rate, calculated by comparing the recovered mass to the theoretical PA6 content based on the 80:20 wool/PA6 ratio, exceeded 80% (Figure S1b). These results collectively demonstrate that the two-step acid process achieves excellent selective separation of PA6 from wool/PA6 blends with minimal wool fiber dissolution.

A visible color change occurs during AcOH pretreatment. As the originally multicolored blended fiber fragments turn uniformly dark brown, the AcOH bath becomes light brown, indicating effective extraction of surface dyes. To assess the decolorization efficiency of dyed wool/PA6 blends using AcOH, two monochromatic fiber samples dyed with C.I. Acid Blue 317 and C.I. Acid Black 207 were subjected to identical pretreatment conditions (Table S1). For the blue-dyed fiber, treatment resulted in a shift to dark gray, and the solution turned deep blue, confirming efficient desorption of the dye from the fiber matrix. In contrast, the black-dyed fibers retained their dark color, with only a faint purple hue observed in the solution. This discrepancy arises because AcOH, as a mild organic solvent, primarily disrupts weak interfacial interactions (e.g., physical adsorption, hydrogen bonding, ionic interactions) between dyes and fiber matrices [32]. It is incapable of cleaving the stronger bonds associated with high-fastness Acid Black 207, which features a larger conjugated structure that allows deeper penetration into the fiber bulk and more stable binding to macromolecular chains [33].

**Figure 1:**
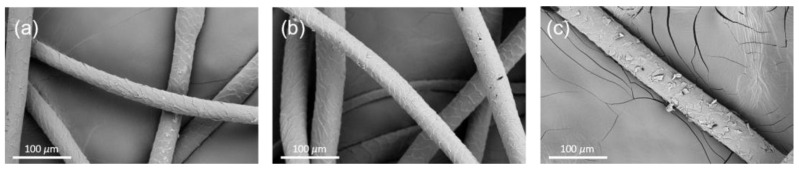
SEM images of (**a**) virgin wool fibers, (**b**) wool fibers dyed with C.I. Acid Blue 317 after AcOH pretreatment and HCOOH dissolution, and (**c**) wool fibers dyed with C.I. Acid Blue 317 after treatment with a thiourea dioxide/SDS aqueous mixture and HCOOH.

Given the limited effectiveness of AcOH in removing high-fastness dark dyes, two literature-reported pretreatment systems [34,35,36], including a AcOH/chlorobenzene (1:1, *v*/*v*) and thiourea dioxide/sodium dodecyl sulfate (SDS) mixture (mass ratio of 1:3), were also evaluated (Table S1). Unfortunately, these improved decolorization methods resulted in significant fiber damage. A representative example occurred with the blue-blended fibers; treatment with AcOH/chlorobenzene transformed the color to brown, while the wool component experienced irreversible damage, losing its original fibrous morphology. After drying, the fibers took on a flat-cake appearance (as shown in Table S1). SEM characterization further confirmed that the thiourea dioxide/SDS mixture severely eroded the outer scale layer of wool fibers (Figure 1c), making the fiber surface visibly rougher. This structural damage is attributed to solvent swelling combined with chemical and thermal stresses, which accelerate deterioration of the wool fiber surface under acidic conditions and likely contribute to structural collapse [37,38]. In comparison, wool treated with AcOH and HCOOH retained a structure consistent with virgin wool (Figure 1a,b), indicating that these two weak acids did not cause significant corrosion to the wool fiber surface.

**Figure 2:**
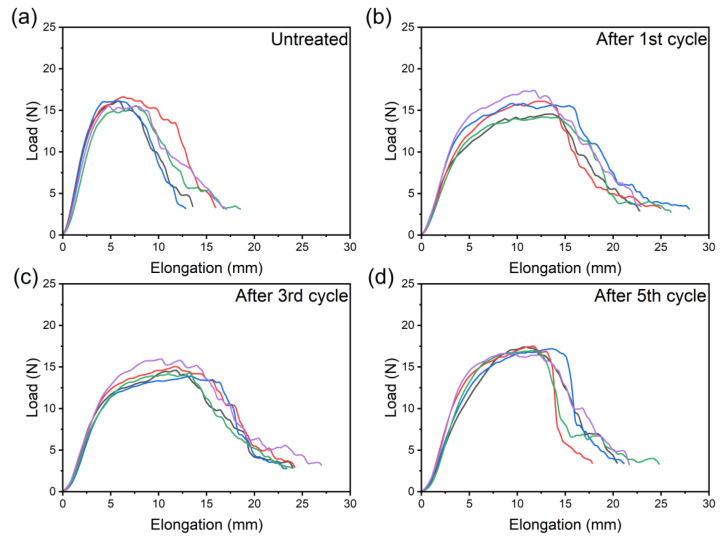
Tensile load–elongation curves for wool yarns subjected to various treatment cycles: (**a**) untreated, (**b**) after 1 cycle, (**c**) after 3 cycles, and (**d**) after 5 cycles. Curves in different colors correspond to the test results of five replicate specimens for each group.

To further confirm that the structure of isolated wool fibers remained intact, tensile tests were conducted on wool yarns (10 cm gauge length) subjected to the two-step acid treatment for 1, 3, and 5 cycles. The representative load–elongation curves of each group are shown in Figure 2, and the quantitative statistical results of tensile properties are summarized accordingly. The results indicate that the tensile properties of wool yarns, in terms of peak load and breaking elongation, showed no significant difference across different treatment cycles compared with virgin wool yarns. The peak load of untreated virgin wool yarns was 15.6 ± 0.5 N, and the breaking elongation was 7.7 ± 1.5 mm. After one treatment cycle, the overall load-bearing capacity of the yarns remained comparable to that of the virgin samples, with a peak load of 15.6 ± 1.3 N (most specimens achieving a peak load of 14–18 N). Meanwhile, the breaking elongation showed a slight increase compared with the untreated group (13.6 ± 1.4 mm). After 3 treatment cycles, the breaking elongation of the yarns was further slightly increased to 13.9 ± 1.5 mm, while the peak load remained at 14.3 ± 0.6 N. This change can be attributed to enhanced fiber wettability and inter-fiber lubrication, as the acidic solution penetrates inter-fiber gaps and wets fiber surfaces during the process. Even after five consecutive treatment cycles, neither the tensile load-bearing performance nor the breaking elongation showed statistically significant differences from virgin wool yarns. These findings confirm that the mild two-step acid treatment imposes negligible damage to the mechanical properties of wool fibers, and their mechanical performance can be well preserved even after repeated treatment cycles.

**Figure 3:**
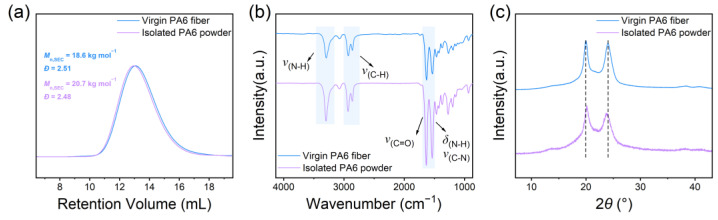
(**a**) Evolution of SEC traces, (**b**) FTIR spectra, and (**c**) XRD patterns of virgin PA6 fiber and recovered PA6 powder obtained via acid-assisted dissolution and reprecipitation.

In addition to the integrity of the recovered wool fibers, the structural properties of the recovered PA6 were systematically characterized to evaluate the influence of the dissolution–reprecipitation process. Characterization was performed using Size exclusion chromatography (SEC), Fourier transform infrared spectroscopy (FTIR), and X-ray diffraction (XRD) measurements (Figure 3). SEC traces revealed that the isolated PA6 powder exhibited a slightly higher number-average molar mass (*M*_n,SEC_) and a narrower molar mass distribution (*Đ*) compared with virgin PA6 fiber, suggesting that the treatment process does not induce degradation of the PA6 molecular chains. This minor increase in molar mass is reasonably attributed to fractionation during dissolution and reprecipitation. The FTIR spectrum of recovered PA6 displayed all the characteristic absorption bands found in virgin PA6, including *N*–*H* stretching (~3300 cm^−1^), *C*=*O* stretching (~1635 cm^−1^), and coupled *N*–*H* in-plane bending and *C–N* stretching (1540 cm^−1^) [39], with no new peaks or significant shifts observed. XRD analysis revealed that virgin PA6 exhibited characteristic diffraction peaks at 2θ = 20.0° and 24.0°, corresponding to the (200) and (002/202) planes of the *α* crystalline phase, respectively [40]. In the recovered PA6, the (200) reflection shifted slightly to a higher angle (20.2°), while the intensity of the (002/202) reflection decreased. This phase transition, typically induced by hydrothermal or dissolution–recrystallization processes, is consistent with the dissolution and reprecipitation methods employed, leading to improved chain packing without altering the overall polymorph [41].

After that, the cyclic stability and long-term reliability of the two-step acidic separation process were evaluated by performing five consecutive separation–recovery cycles. As shown in Figure S2, the recovery rates for both acids were at a relatively high level of about 90% in the first cycle. As the number of cycles increased, the solvent recovery rates gradually declined, primarily due to unavoidable solvent evaporation during hot filtration and sample transfer operations. These losses can be further reduced through process optimization (e.g., adopting a closed-loop operating configuration). Notably, throughout the five cycles, the recovery rates for both acids consistently remained above 80%, demonstrating that this approach can effectively achieve solvent recovery and reuse, thereby offering practical value. Furthermore, when the recovered acid solution was used for subsequent separation, the PA6 recovery rate remained stable at over 75%, and the mass of the separated wool fibers showed no significant change. This indicates that this separation method exhibits good long-term operational stability.

### 3.2. Microwave-Assisted Depolymerization of Recovered PA6

While the recovered PA6 powder can be directly melt-processed into secondary products, physical recycling typically results in cumulative molecular degradation and contamination over multiple cycles. These detrimental effects progressively diminish the overall performance of recycled polymers, ultimately limiting their service life. Thus, monomer-level regeneration is essential for achieving complete closed-loop recycling of PA6 and restoring its intrinsic properties. Inspired by previously reported microwave-mediated polymer degradation techniques [27], we employed a microwave-assisted heating strategy in conjunction with an acetic anhydride/organobase catalytic system for the efficient depolymerization of recovered PA6 into AcCL. Microwave heating is favored over traditional heating methods due to its ability to enhance reaction kinetics and reduce depolymerization time. Organobases are selected as catalysts due to their low toxicity and absence of metallic residues, facilitating an environmentally friendly depolymerization process.

**Figure 4:**
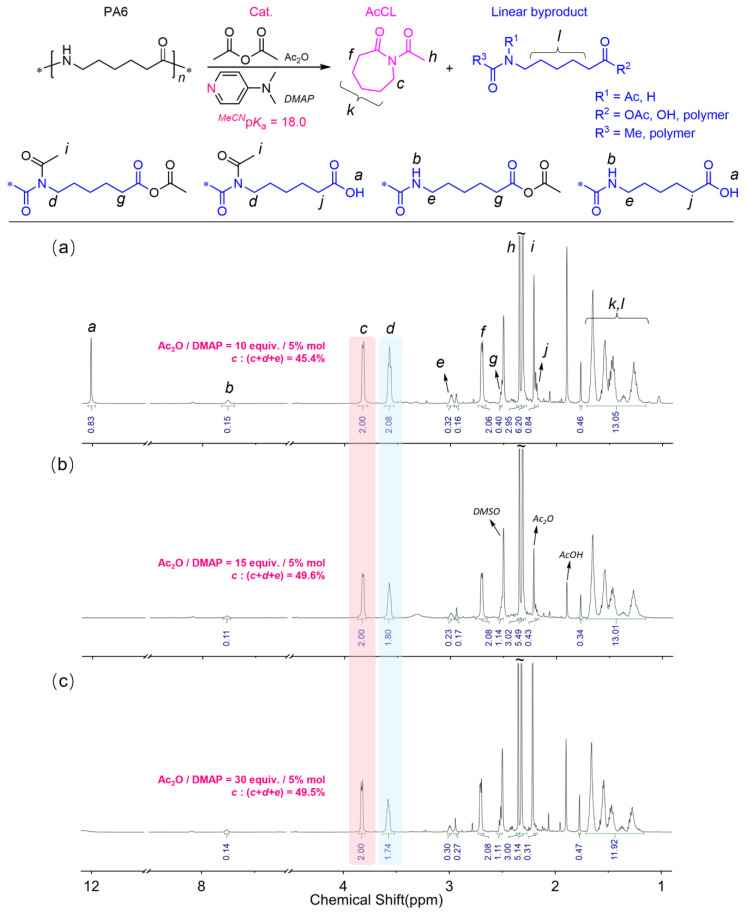
(**Upper**): Depolymerization of PA6 to *N*-acetyl-*ε*-caprolactam (AcCL) catalyzed by the DMAP/Ac_2_O system, alongside the chemical structures of the resulting linear oligomeric byproducts. (**Lower**): (**a**–**c**) ^1^H NMR spectra (DMSO-*d*_6_) of depolymerization products from (**a**) Entry 1 (10 equiv. Ac_2_O), (**b**) Entry 2 (15 equiv. Ac_2_O), (**c**) Entry 3 (30 equiv. Ac_2_O), all with 5 mol% DMAP.

The conditions and results of the depolymerization experiments are summarized in Table 1. Initial experiments were conducted using 4-dimethylaminopyridine (DMAP) as a model catalyst to investigate the effect of Ac_2_O loading on AcCL yield (Entries 1–3). No solid residue was observed post-reaction, indicating complete degradation of PA6 into soluble products through amide bond cleavage and intramolecular cyclization. ^1^H NMR spectra of the depolymerization products (Figure 4) exhibit a characteristic resonance at ~3.8 ppm (peak *c*) corresponding to cyclic AcCL, as well as signals at ~3.5 and ~3.0 ppm (peaks *d* and *e*) corresponding to linear oligomeric byproducts. Based on these spectral assignments, the AcCL yields were quantified as 45.4%, 49.6%, and 49.5% for Ac_2_O loadings of 10, 15, and 30 equivalents, respectively.

To elucidate the intrinsic role of Ac_2_O in the depolymerization process, structural analyses of the cyclic AcCL and the linear byproducts were conducted. As a highly effective acylating agent, Ac_2_O readily reacts with the backbone amide groups of PA6, forming acetimide structures, while unreacted amide groups remain largely undetected (peak *b*). Increasing the dosage from 10 to 15 equivalents enhanced the proportion of cyclic AcCL and diminished the presence of linear oligomers. This improvement is attributed to the *N*-acetylation of the amide bonds, resulting in highly reactive intermediates that facilitate intramolecular backbiting for AcCL formation. A weak carboxylic proton signal near 12 ppm can be attributed to trace water-induced amide hydrolysis, and its gradual disappearance with increasing Ac_2_O loading indicates the chain-end capping capacity of acetic anhydride. By terminating free amine and carboxyl terminals in oligomers, Ac_2_O effectively suppresses undesirable side reactions, thereby promoting the formation of cyclic products and increasing AcCL yield. However, an excessive Ac_2_O dosage did not yield further improvements in cyclization selectivity.

**Figure 5:**
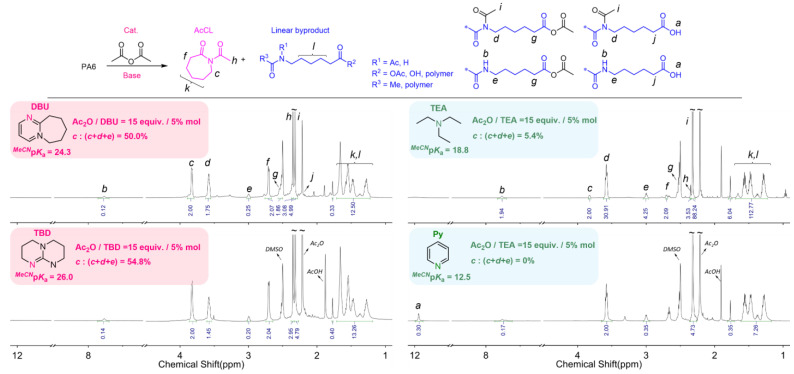
(**Upper**): Reaction pathway for PA6 depolymerization to AcCL using Ac_2_O with various organobases. (**Lower**): ^1^H NMR spectra (DMSO-*d*_6_) of products from Entries 5–8.

**Figure 6:**
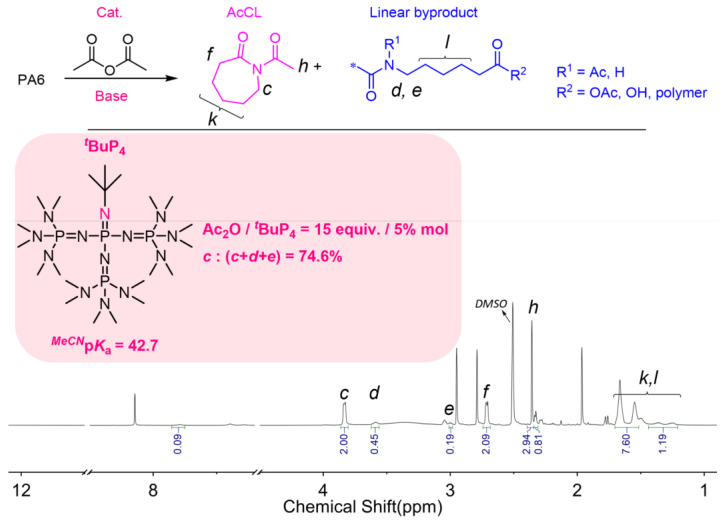
(**Upper**): Reaction pathway for PA6 depolymerization to AcCL catalyzed by Ac_2_O/*^t^*BuP_4_. (**Lower**): ^1^H NMR spectra (DMSO-*d*_6_) of products from Entry 9.

A base-free control experiment using 15 equiv. of Ac_2_O (Entry 4, Figure S3) yielded 40.3% AcCL, lower than the results observed in DMAP-catalyzed reactions. This finding corroborates the effectiveness of organobases in promoting intramolecular cyclization during PA6 depolymerization. To systematically explore the relationship between catalyst basicity and cyclization selectivity, a series of organobases exhibiting varying basicity was screened with a fixed loading of 15 equiv. of Ac_2_O (Figure 5). Two moderately stronger bases, DBU (p*K*_a_ = 24.3 in *MeCN*) and TBD (p*K*_a_ = 26.0 in *MeCN*), exhibited higher cyclization selectivity than DMAP, yielding AcCL amounts of 50.0% and 54.8%, respectively. Further increasing the basicity with the superbasic phosphazene base *^t^*BuP_4_ (p*K*_a_ = 42.7 in *MeCN*) resulted in a maximum AcCL yield of 74.6% (Figure 6). This trend demonstrates that stronger bases promote cyclization by abstracting *N*–*H* protons from PA6 amide bonds, generating reactive amide anion intermediates, which are essential for cyclic monomer formation. In contrast, the weakest base examined, pyridine (Py, p*K*_a_ = 12.5 in *MeCN*), completely failed to catalyze the formation of cyclic AcCL, further corroborating that sufficient basicity is indispensable for cyclization.

However, this monotonic correlation does not extend to all organobases, as evidenced by the striking disparity between DMAP and triethylamine (TEA). Despite TEA (p*K*_a_ = 18.8 in *MeCN*) being slightly more basic than DMAP (p*K*_a_ = 18.0 in *MeCN*), the cyclization selectivity it affords is drastically lower, producing only 5.4% AcCL versus 49.6% for DMAP. More importantly, both TEA and Py actually inhibited the cyclization relative to the base-free control (yielding 40.3% of AcCL), resulting in even lower yields. This pronounced departure from the basicity-selectivity trend indicates that basicity alone cannot fully account for the observed cyclization performance.

**Scheme 2:**
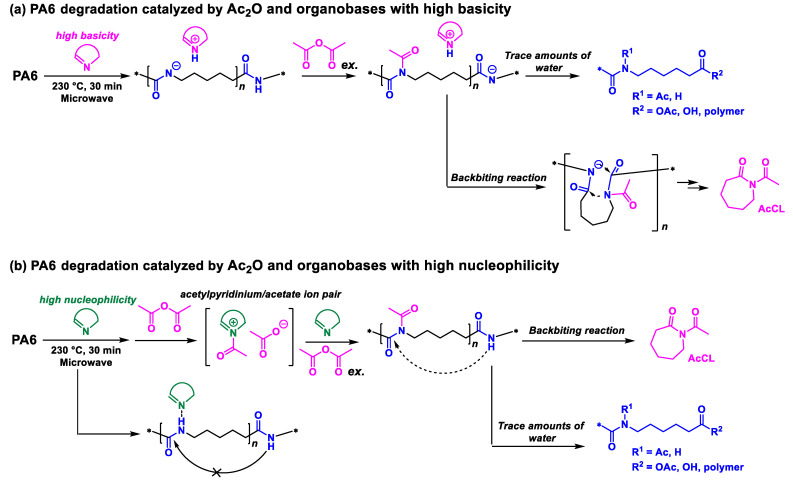
Proposed mechanistic pathways for the depolymerization of PA6 to AcCL using Ac_2_O with organobases of varying basicity and nucleophilicity.

The distinct catalytic performances among different organobases arise from the dual functional properties of base catalysts, coupled with two competing reaction pathways in the Ac_2_O-mediated PA6 depolymerization system (Scheme 2). High-basicity, low-nucleophilicity catalysts such as *^t^*BuP_4_ facilitate the reaction through an efficient deprotonation-coupled intramolecular backbiting cyclization pathway, in which efficient abstraction of amide *N*–*H* protons generates highly nucleophilic amide anions that predominate and enable high AcCL production. In this mechanism, acetic anhydride serves a dual function, acting as both an activator of acetimide formation and an effective chain-end capping agent. The observed increase in AcCL yield and selectivity with rising catalyst basicity robustly supports this proposed cyclization mechanism. In contrast, DMAP operates through a mechanistically distinct pathway that exploits its strong nucleophilicity in combination with moderate basicity. It is proposed that DMAP reacts rapidly with Ac_2_O to generate a stable, highly reactive acetylpyridinium intermediate, which can directly mediate *N*-acetylation of PA6 amide groups. This acetyl transfer process significantly improves both the yield and selectivity of AcCL, consistent with the well-established role of DMAP in alcohol acetylation [43].

Conversely, TEA and Py cannot efficiently operate via either pathway. TEA, although slightly more basic than DMAP, has negligible nucleophilicity and thus cannot form the required acetylpyridinium-type intermediate. Meanwhile, its moderate basicity is insufficient to generate a steady-state concentration of amide anions high enough to render the deprotonation–cyclization pathway productive. Py, which has the lowest basicity in the series, is even less effective at deprotonating the amide bonds, and it also fails to form an active acylating species. Consequently, neither TEA nor Py can promote the cyclization pathway to any significant extent. Instead, their presence appears to accelerate competing hydrolytic side reactions, as evidenced by the detection of carboxy-terminated oligomers in the Py-catalyzed system. This diversion of the substrate toward hydrolysis rather than cyclization accounts for the fact that the AcCL yields obtained with TEA and Py are both substantially lower than that of the base-free control. Additionally, reaction temperature was found to be a critical parameter affecting depolymerization efficiency (Entries 10–11). At an excessively low temperature of 160 °C, PA6 was not completely dissolved (Entry 10, Figure S4), which hindered efficient depolymerization. In contrast, increasing the temperature to 260 °C caused severe thermal decomposition and the formation of black, insoluble carbonaceous byproducts, with no detectable cyclic products obtained (Entry 11, Figure S5). This narrow optimal temperature range highlights the necessity for precise temperature control during microwave-assisted depolymerization.

### 3.3. Closed-Loop Monomer Regeneration of PA6

**Figure 7:**
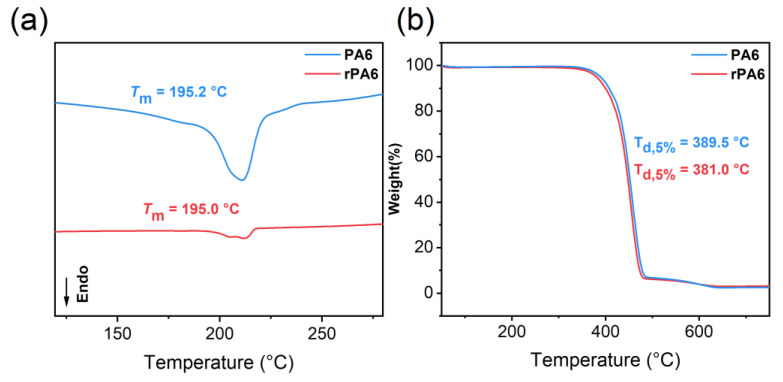
(**a**) DSC (second heating curves) and (**b**) TGA curves of virgin PA6 and rPA6.

The AcCL generated from depolymerization was subsequently deacetylated via transamidation with MEA, producing high-purity CPL (Figure S6) [27]. The regenerated CPL underwent repolymerization using the same *^t^*BuP_4_ superbase catalyst. This integrated catalytic approach streamlines the overall process and enhances the reproducibility of the recycling system, a benefit directly attributable to the well-established recoverability and reusability of phosphazene bases [44,45]. The chemical structure and properties of the regenerated PA6 (rPA6) were characterized and compared to those of virgin PA6. SEC analysis indicated that rPA6 exhibits a molar mass of 15.9 kg mol^−1^ with unimodal distribution (*Ð* = 2.06; Figure S7), suggesting the high efficiency of the *^t^*BuP_4_ catalyst for CPL polymerization and the exceptional purity of the regenerated monomer. FTIR spectroscopy confirmed that rPA6 has the same chemical structure as virgin PA6, with all characteristic amide absorption bands appearing at the same wavenumbers. The thermal properties of the polymers were further assessed through differential scanning calorimetry (DSC) and thermogravimetric analysis (TGA). DSC analysis revealed that rPA6 has a melting temperature (*T*_m_) of 195.0 °C, closely aligning with the 195.2 °C observed for virgin PA6. TGA results showed that rPA6 maintains favorable thermal stability, with the temperature for 5% weight loss (*T*_d,5%_) of 381.0 °C, comparable to the 389.5 °C for virgin PA6 (Figure 7).

## 4. Conclusions

In summary, this study presents a mild acid-assisted closed-loop recycling strategy for efficient component separation and high-value PA6 regeneration from waste carpet fibers. AcOH pretreatment provides mild decolorization of the blended fibers, while HCOOH enables selective dissolution and high-purity recovery of PA6. Both acids are recyclable via distillation for cyclic operation. Over five consecutive separation–recovery cycles, the recovery rates of both acid solvents remained above 80%, and the PA6 recovery yield in each cycle consistently exceeded 75%. Characterization results confirm that the recovered PA6 retains its chemical structure, and the separated wool fibers preserve their original fibrous morphology and tensile properties. Building on this, the recovered PA6 is further depolymerized via a microwave-assisted acetic anhydride/organobase system. Using the phosphazene base *^t^*BuP_4_ as the catalyst, a maximum AcCL yield of 74.6% is attained. After deacetylation, the resultant AcCL is converted to CPL, which subsequently undergoes ring-opening polymerization also catalyzed by *^t^*BuP_4_, ultimately realizing monomer-level closed-loop recovery and regeneration of PA6. The entire recycling process operates under mild, metal-free conditions with reusable solvents, and microwave heating reduces overall energy consumption, demonstrating strong potential for industrial-scale deployment.

## Figures and Tables

**Table 1:** Conditions and Results for the depolymerization of recovered PA6 *^a^*.

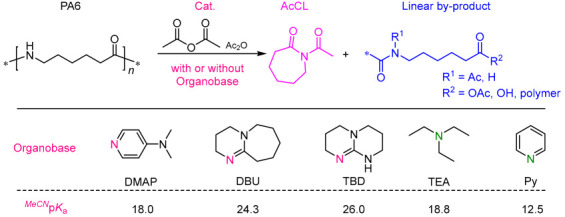				
Entry	Base	Base/Ac_2_O *^b^*	Temp. (°C) *^c^*	Yield of AcCL (%) *^d^*
1	DMAP	5 mol%/10	230	45.4
2	DMAP	5 mol%/15	230	49.6
3	DMAP	5 mol%/30	230	49.5
4	- *^e^*	-/15	230	40.3
5	DBU	5 mol%/15	230	50.0
6	TBD	5 mol%/15	230	54.8
7	TEA	5 mol%/15	230	5.4
8	Py	5 mol%/15	230	- *^f^*
9	*^t^*BuP_4_	5%/15	230	74.6
10	*^t^*BuP_4_	5%/15	160	- *^g^*
11	*^t^*BuP_4_	5%/15	260	- *^h^*

*^a^* Depolymerization experiments employed 0.10 g of recovered PA6 powders as the starting material and were conducted in a sealed microwave reactor for 30 min, with pressure monitored to remain below 4 MPa. The p*K*_a_ values for all bases were obtained from the literature [42]. *^b^* Catalyst dosage is specified based on the PA6 repeating unit. *^c^* Reactions were performed at the designated temperature. *^d^* AcCL yield was determined by ^1^H NMR using deuterated dimethyl sulfoxide (DMSO-*d*_6_) as the solvent, with molar fractions calculated via peak area integration, reflecting the selectivity of AcCL relative to linear oligomers. *^e^* No base catalyst was utilized. *^f^* Not detected. *^g^* PA6 was not completely dissolved. *^h^* Black insoluble by-products were generated.

## Data Availability

The original contributions presented in this study are included in the article/Supplementary Materials. Further inquiries can be directed to the corresponding authors.

## Supplementary Materials

The following supporting information can be downloaded at https://www.mdpi.com/article/10.3390/polym18151800/s1, Table S1: Decolorization efficiency of dyed wool/PA6 blended fibers under different pretreatment conditions; Figure S1: (a) Mass retention of pure PA6 and wool fibers after AcOH treatment; (b) Recovered mass of PA6 and retained mass of wool separated from blended fibers via the two-step acid treatment; Figure S2: Recovery rates of (a) AcOH, (b) HCOOH, (c) PA6 powder, and (d) wool fibers over five consecutive cycles; Figure S3: Upper: Schematic illustration of depolymerization of PA6 catalyzed by Ac_2_O. Lower: ^1^H NMR spectrum (DMSO-*d*_6_) of the isolated product of entry 4; Figure S4: Upper: Schematic illustration of depolymerization of PA6 catalyzed by Ac_2_O and *^t^*BuP_4_ at 160 °C. Lower: ^1^H NMR spectrum (DMSO-*d*_6_) of the isolated product of entry 10; Figure S5: Upper: Schematic illustration of depolymerization of PA6 catalyzed by Ac_2_O and *^t^*BuP_4_ at 260 °C. Lower: ^1^H NMR spectrum (DMSO-*d*_6_) of the isolated product of entry 11;. Figure S6. Upper: Schematic illustration of the deacetylation of AcCL to obtain purified CL. Lower: ^1^H NMR spectrum (DMSO-*d*_6_) of the isolated AcCL and CL; Figure S7: (a) Evolution of SEC traces and (b) FTIR spectra of rPA6.
