# Supplementary material for: Mild Acid-Assisted Separation and Closed-Loop Monomer-Level Regeneration of Polyamide 6 from Waste Wool/PA6 Carpet Blends
Source: Polymers (Basel). 2026 Jul 23;18(15):1800. doi: 10.3390/polym18151800 (PMC13468513; doi:10.3390/polym18151800)
Supplement: Supplementary file 1 [file polymers-18-01800-s001.zip › polymers-4398113-supplementary.pdf]

Supporting Information

Mild Acid-Assisted Separation and Closed-Loop Monomer-Level Regeneration of polyamide 6 from Waste Wool/PA6 Carpet Blends

Guohu Wang[1,#], *Shimu Yu*[1,#], *Fengzhuang Liu*[1], *Guodong Liu*[1], *Chengsheng Zhang*[2,*], *Hongxin Zhang*[1,*]

[1] School of Chemical Engineering, Qinghai University, Xining 810016, People's Republic of China

[2] Shengyuan Carpet Group Co., Ltd., Xining 810016, People's Republic of China

* Correspondence: zcs.job@163.com (CS.Z.); mszh.xin@qhu.edu.cn (HX.Z.)

Table of content

Table S1......3

Figure S1......4

Figure S2......4

Figure S3......5

Figure S4......6

Figure S5......7

Figure S6......8

Figure S7......8

Table S1. Decolorization efficiency of dyed wool/PA6 blended fibers under different pretreatment conditions [a]

Dyes type	Pretreatment solvents	Before pretreatment	After pretreatment	Solution after treatment
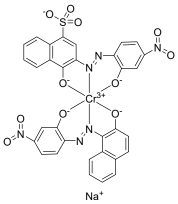 C.I. Acid Blue 317	AcOH	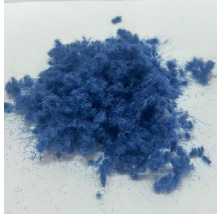	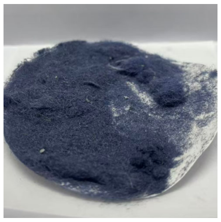	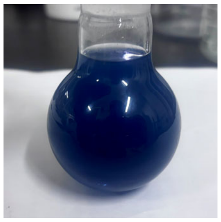
	AcOH/ chlorobenzene	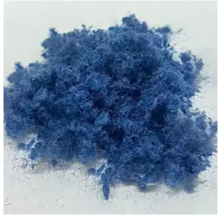	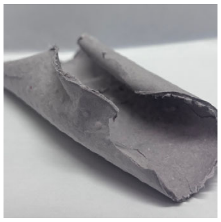	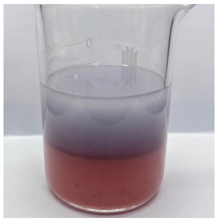
	Thiourea dioxide/ SDS aqueous solution	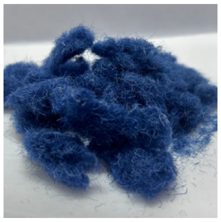	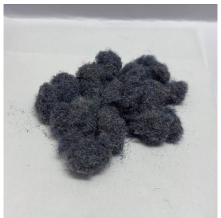	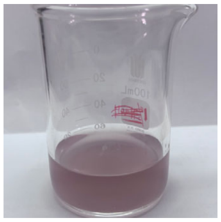
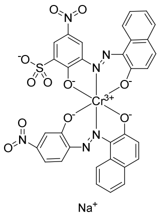 C.I. Acid Black 207	AcOH	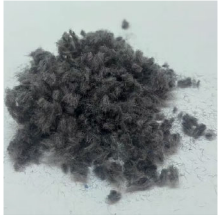	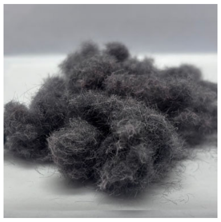	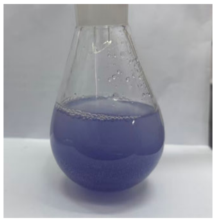
	AcOH/ chlorobenzene	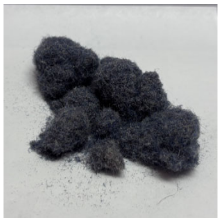	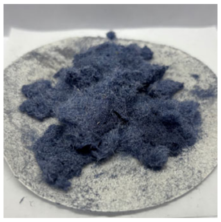	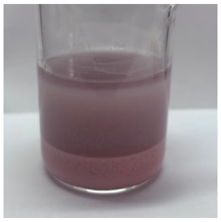
	Thiourea dioxide/ SDS aqueous solution	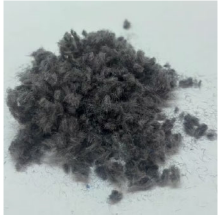	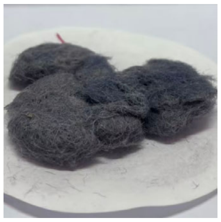	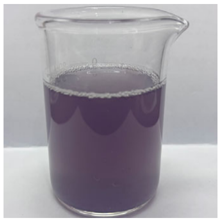

[a] All pretreatments were conducted at 80 °C for 5 h with a liquor-to-fiber ratio of 1:30. Two types of monochromatic dyed wool/PA6 blended fibers (blue and black) were used in this part.

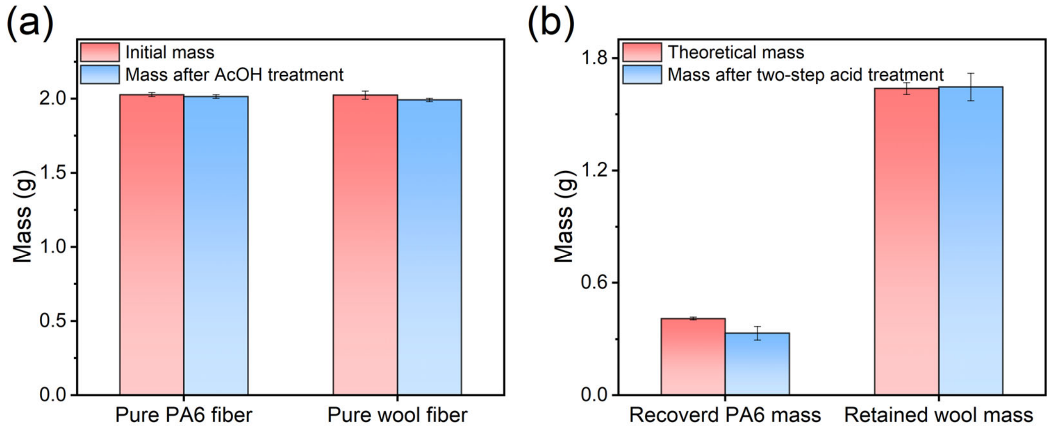

Figure S1.(a) Mass retention of pure PA6 and wool fibers after AcOH treatment; (b) Recovered mass of PA6 and retained mass of wool separated from blended fibers via the two-step acid treatment.

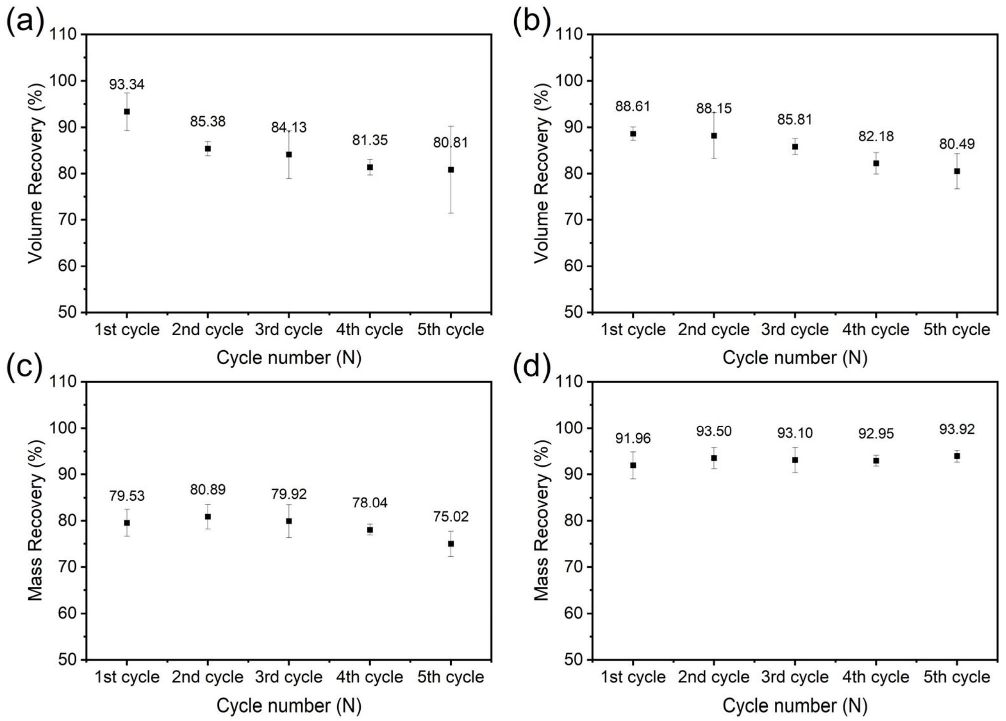

Figure S2. Recovery rates of (a) AcOH, (b) HCOOH, (c) PA6 powder, and (d) wool fibers over five consecutive cycles.

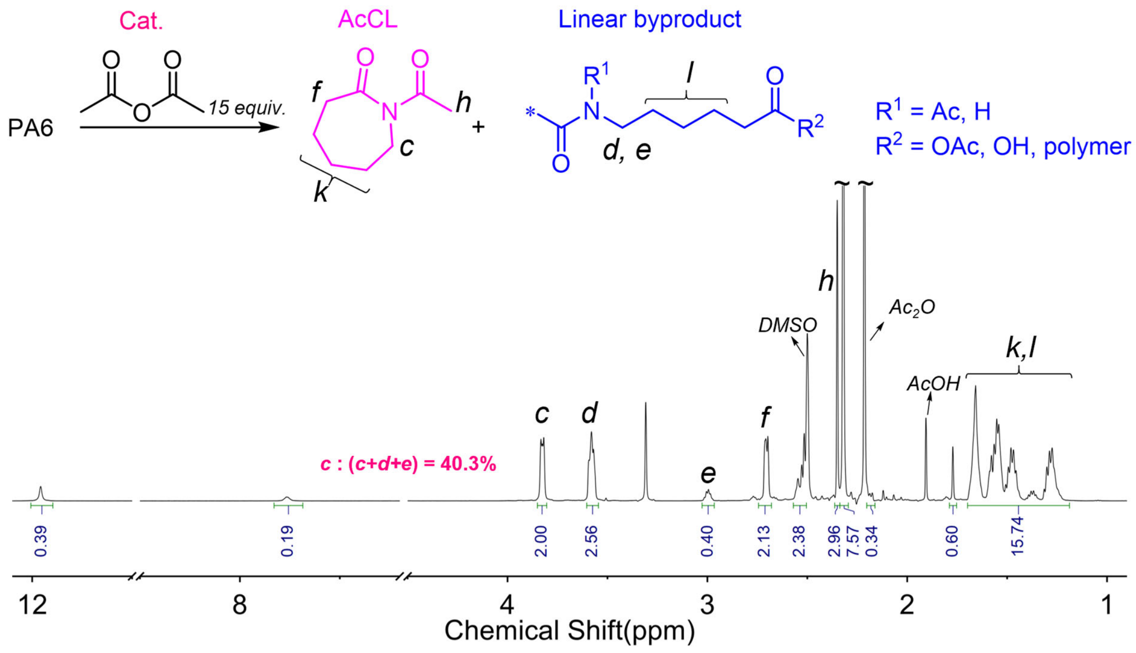

Figure S3. Upper: Schematic illustration of depolymerization of PA6 catalyzed by Ac_2O. Lower: ^1H NMR spectrum (DMSO-d_6) of the isolated product of entry 4.

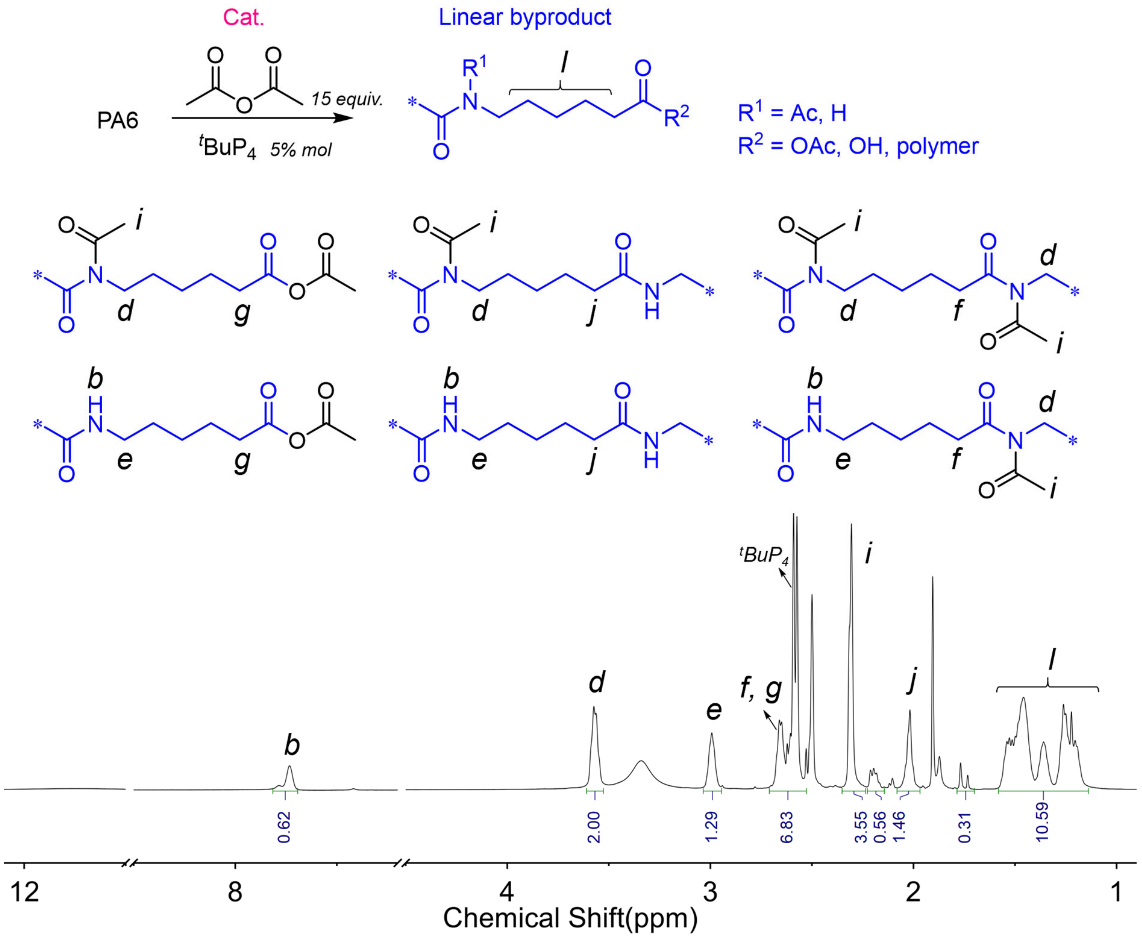

Figure S4. Upper: Schematic illustration of depolymerization of PA6 catalyzed by Ac_2O and $^tBuP_4$ at 160 °C. Lower: ^1H NMR spectrum (DMSO-d_6) of the isolated product of entry 10.

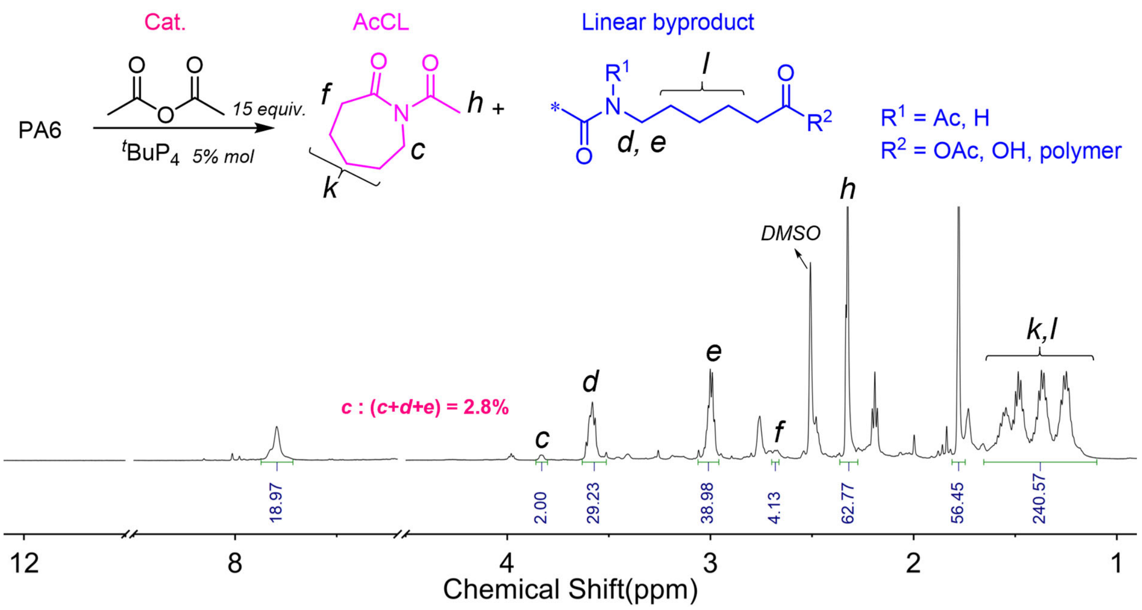

Figure S5. Upper: Schematic illustration of depolymerization of PA6 catalyzed by Ac_2O and ^{t}BuP$_4$ at 260 °C. Lower: ^{1}H NMR spectrum (DMSO-d_6) of the isolated product of entry 11.

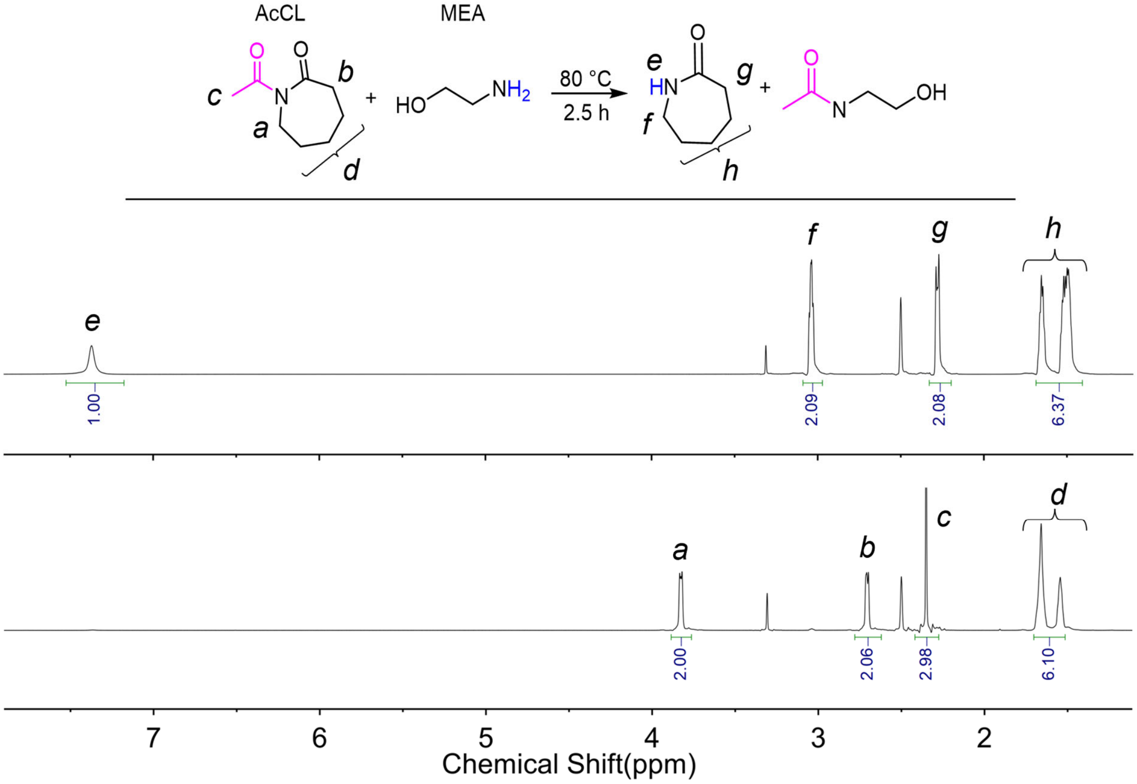

Figure S6. Upper: Schematic illustration of the deacetylation of AcCL to obtain purified CL. Lower: ^{1}H NMR spectrum (DMSO-d_6) of the isolated AcCL and CL.

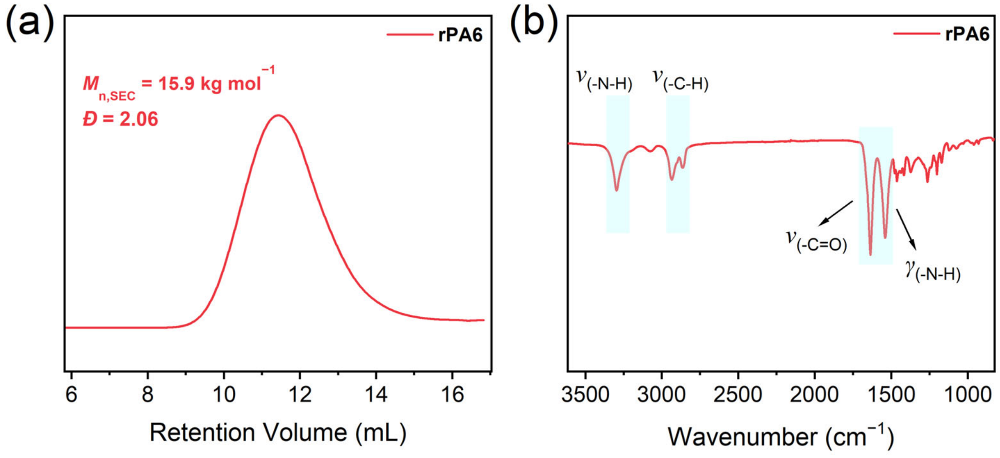

Figure S7.(a) Evolution of SEC traces and (b) FTIR spectra of rPA6.
